# Serial head circumference measurements should be used to classify congenital microcephaly

**DOI:** 10.1186/s12887-023-04315-4

**Published:** 2023-09-27

**Authors:** Chutima Sengasai, Kulkanya Chokephaibulkit, Nottasorn Plipat, Pimol Wongsiridej

**Affiliations:** 1 Department of Pediatrics, Faculty of Medicine Siriraj Hospital, Mahidol University, 2, Wanglang Road, Bangkok-Noi District, Bangkok, 10700 Thailand; 2 Siriraj Institute of Clinical Research, Faculty of Medicine Siriraj Hospital, Mahidol University, 2, Wanglang Road, Bangkok-noi District, Bangkok, 10700 Thailand

**Keywords:** Head circumference, Thailand, Neonates, Newborn infants, Microcephaly

## Abstract

**Background:**

Measuring the maximum occipitofrontal circumference only once at birth or within 24 h after birth may lead to misclassifications of microcephaly. This study compared the head circumference (HC) of newborns at birth or within 24 h after birth to their third day of life (DOL3) as well as evaluated maternal- and infant-specific factors associated with increased HC by DOL3.

**Methods:**

This prospective study included 1131 live births between February and May 2019 with a gestational age > 27 weeks. All newborns had their HC measured at birth or within 24 h after birth as well as on DOL3 before discharge. HC measurements were performed by trained personnel using non-elastic tape measures. The World Health Organization (WHO) and Fenton Growth Charts were used as reference ranges for interpretation of full-term and preterm neonates, respectively.

**Results:**

Paired sample t-test analyses found a statistically significant increase in HC measured on the DOL3 compared with HCs of the same newborns at birth or within 24 h of birth. The mean HC increase was 0.17 cm (95% confidence interval [0.13, 0.21], *P* < 0.001). The mean ± standard deviation HC within 24 h of birth and at DOL3 were 33.58 ± 1.53 cm and 33.75 ± 1.37 cm, respectively. Thirty-two newborns had HCs less than the third percentile (< P3) at birth, 25 of which had HC ≥ P3 at DOL3. After adjusting for mode of and presentation at delivery, newborns whose mothers experienced labor pains (β = 0.31, *P* < 0.001) and were either symmetrically (β = 0.59, *P* = 0.002) or asymmetrically small-for-gestational age (SGA; β = 0.37, *P* = 0.03) had significantly increased HC at DOL3. On average, newborns whose mothers experienced labor pain had 0.31 cm increases in HC at DOL3. Symmetrical SGA newborns also had an average 0.59 cm increase in HC at DOL3. Parity and gestational age were not associated with changes in HC.

**Conclusions:**

Serial HC measurements on DOL3 or before newborns’ discharge is crucial to classifying congenital microcephaly.

## Background

Microcephaly in neonates is defined by a maximum occipitofrontal circumference greater than 2 standard deviations (SDs) below the mean (less than the third percentile [< P3]). While performing head circumference (HC) measurements are simple, the results can be mismeasured. Despite standardization efforts, HC measurements are not always accurate due to technical errors – even after training [1].

The delivery process itself can also introduce anthropometric changes to newborns’ head size at birth. Skull molding during vaginal delivery may reduce head size while caput succedaneum, cephalhematomas, and subdural hematomas may increase it [2, 3]. These anthropometric changes may take up to a week or more to resolve. However, congenital microcephaly is often classified based on initial measurements within 24 h after birth per the recommendations of the World Health Organization (WHO) and United States Centers for Disease Control and Prevention (CDC) [4, 5]. As a result, over- and underreporting of microcephaly is common, subsequently leading to unnecessary, costly investigations and misclassifications.

This study assessed the difference in HC measured at birth or within 24 h of birth to the third day of life (DOL3) for the same newborns. It also assessed maternal- and infant-specific factors that may impact differences in HC. The researchers hypothesized that repeated HC measurements on DOL3 would differ from HC measured at birth, highlighting the importance of serial measurements to accurately classify microcephaly.

## Methods

### Study design and participants

This prospective study was conducted at Siriraj Hospital – the largest public quaternary care medical center in Bangkok, Thailand with a capacity of more than 2000 beds and 7000 deliveries annually. Live births at a gestational age > 27 weeks between February and May 2019 were included in the study. Infants referred to other hospitals earlier than DOL3 and neonatal deaths within 24 h after birth were excluded. This study was approved by the Research Ethics Board at Siriraj Hospital (Si 650/2018) and performed per the International Council on Harmonization’s Good Clinical Practice, Belmont Report, and Declaration of Helsinki. All newborns were enrolled after parents provided written informed consent.

### Study procedures

All newborns had their HC measured soon after delivery by nursing staff at one of the following delivery units: common or private labor rooms, septic labor room, or caesarean section (C-section) room. Twenty nurses from these units participated in in-person training sessions held by one of the authors (C.S.). Intraclass correlation coefficients (ICC) for inter- and intra-observer reliabilities were examined in 15 out of 21 participants. The results confirmed highly reliable measurements (ICCs of 0.991 and 0.998, respectively).

Maximum occipitofrontal circumference was measured thrice (with the largest number of the three measures being recorded) using non-elastic tape measures at birth or within 24 h of birth as well as on DOL3 before newborns were discharged.

### Measured outcomes

All data were recorded while mothers and infants were admitted at Siriraj. Assessed maternal variables included: age; parity; past medical history; prenatal and birth history (i.e., mode of delivery, delivery presentation); cigarette, alcohol, and drug use; as well as laboratory results. Assessed infant variables included: gestational age; birth weight and length; HC; as well as the date and time HC measurements were performed. Gestational age was calculated based on the mother’s last menstrual period (LMP). For mothers who were uncertain about their LMP, gestational age was estimated using an ultrasound report (if available) or Ballard score. Percentiles for each HC were performed using the WHO and Fenton Growth Charts as references for interpreting data of full-term and preterm neonates, respectively.

### Statistical analysis

Descriptive statistical methods (Pearson’s Chi-square or Fisher’s exact test) were used to analyze categorical data. Paired sample t-tests were used to compare HCs at birth or within 24 h of birth and by DOL3. Factors associated with changes to HC (i.e., gestational age, delivery presentation, mode of delivery, and parity) were analyzed using an analysis of variance. Multiple linear regression was performed to analyze the relationship of these factors towards predicting changes in HC at DOL3. All analyses were performed using a Statistic Package for Social Science in SPSS^®^ (v16.0; IBM^®^ Corporation, Armonk, NY, USA).

## Results

**Fig. 1:**
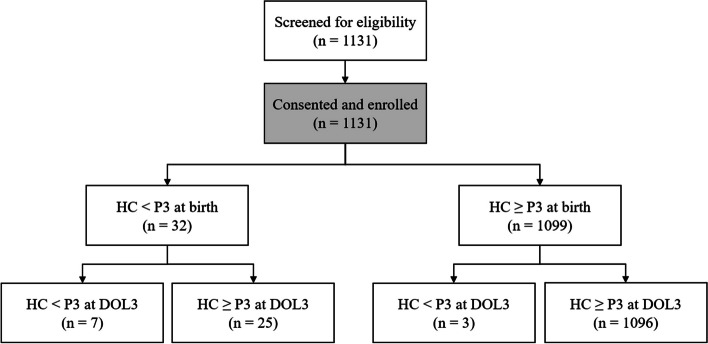
Study flow diagram. The percentiles for HC referenced the WHO for full-term (≥ 37 weeks of gestation) neonates and Fenton Growth Charts for preterm (< 37 weeks of gestation) neonates. HC, head circumference; P3, third percentile; DOL3, 3 days of life

Of 1131 enrolled newborns, 550 (48.6%) were female, and 995 (88.0%) were at term (37–42 weeks gestation). Thirty-two (2.8%) newborns had HCs < P3 at birth while 1099 (97.2%) had HCs ≥ P3 (Fig. 1). The presentations at birth were vertex for 1074 (95.0%), breech for 48 (4.2%), and transverse for 9 (0.9%) newborns. The delivery modes were normal vaginal delivery for 542 (47.9%), vacuum assisted vaginal delivery for 33 (2.9%), forceps assisted vaginal delivery for 4 (0.4%), caesarean section after labor pain for 209 (18.5%), and elective caesarean section for 343 (30.3%) newborns.

A statistically significant increase in HC measured at DOL3 was observed compared with measurements of the same newborns at birth or within 24 h of birth. This mean HC increase was 0.17 cm (95% confidence interval [CI] of [0.13, 0.21], *P* < 0.001). The mean ± standard deviation (SD) HC within 24 h of life and at DOL3 were 33.58 ± 1.53 cm and 33.75 ± 1.37 cm, respectively. Upon assessing the impact of this difference between the two measurements, only seven of 32 newborns with HCs < P3 at birth remained so by the time of discharge. After following up on these seven infants, the researchers found that: three had delayed cognitive development and continued to display microcephaly at 18 months of age; one was normocephalic, but had delayed speech; another was normocephalic and had normal cognitive development; and two were lost to follow-up. Three newborns had been normocephalic at birth but were identified as microcephalic at DOL3 (with a total of ten cases microcephalic at discharge). Of these three newborns, one was diagnosed with congenital cytomegalovirus (CMV) infection while the other two had normal HC and development at subsequent follow-ups. No congenital Zika infections were identified within this study’s cohort.

Among newborns who were microcephalic at birth, vaginal delivery, vertex presentation, labor pain, and being small-for-gestational age (SGA) were factors significantly associated with increased HC at DOL3 (Table 1). After adjusting for mode of and presentation at delivery, newborns whose mother experienced labor pain (average increased HC at DOL3 of 0.31 cm, *P* < 0.001) and who were either symmetrically (average increased HC at DOL3 of 0.59 cm, *P* = 0.002) or asymmetrically (average increased HC at DOL3 of 0.37 cm, *P* = 0.03) SGA had significantly increased HC at DOL3 (Table 2).

**Table 1:** Association between head circumference and maternal and infant factors

	**n**	**Head circumference (cm)**			***P*-value**
		**At birth (HC**_**1**_**)**	**At DOL3 (HC**_**2**_**)**	**Mean difference (HC**_**2**_** - HC**_**1**_**)**	
***Gestation***					
28–< 37 weeks	136	31.89 ± 1.66	32.08 ± 1.57	0.19 ± 0.74	0.712
≥ 37 weeks	995	33.81 ± 1.36	33.98 ± 1.17	0.17 ± 0.76	
***Delivery mode***					
Vaginal	579	33.39 ± 1.42	33.70 ± 1.25	0.32 ± 0.79	< 0.001
Caesarean	552	33.78 ± 0.07	33.80 ± 0.06	0.02 ± 0.03	
***Presentation***					
Vertex	1074	33.56 ± 1.51	33.75 ± 1.34	0.19 ± 1.76	< 0.001
Breech or transverse	57	34.00 ± 1.82	33.83 ± 1.93	-0.16 ± 0.53	
***Labor pain***					
Present	788	33.38 ± 0.05	33.67 ± 0.05	0.29 ± 0.03	< 0.001
Absent	343	34.04 ± 0.08	33.93 ± 0.07	-0.11 ± 0.03	
***Size***					
AGA	1014	33.65 ± 1.43	33.80 ± 1.29	0.15 ± 0.76	< 0.001
Symmetrical SGA	35	31.19 ± 1.72	31.83 ± 1.84	0.64 ± 0.67	
Asymmetrical SGA	54	32.67 ± 1.22	33.00 ± 0.94	0.34 ± 0.72	
LGA	28	35.69 ± 0.99	35.55 ± 1.04	-0.15 ± 0.49	
***Parous***					
G1	487	33.49 ± 1.55	33.71 ± 1.39	0.22 ± 0.75	0.235
G2	422	33.64 ± 1.50	33.76 ± 1.37	0.12 ± 0.72	
G3	164	33.64 ± 1.53	33.82 ± 1.27	0.18 ± 0.87	
G > 3	58	33.77 ± 1.70	33.86 ± 1.58	0.10 ± 0.76	

Data are displayed as mean ± standard deviation and were analyzed using one-way ANOVA

*DOL3* 3 days of life, *SGA* Small-for-gestational age, *LGA* Large-for-gestational age, *AGA* Appropriate-for-gestational age

**Table 2:** Multiple linear regression of changes in head circumference and maternal and infant factors

**Variables**	**Regression Coefficient (β)**	***P*-value**
***Size***		
Symmetrical SGA	0.59	0.002
Asymmetrical SGA	0.37	0.030
AGA	0.14	0.311
LGA	1.00	
***Delivery mode***		
Vaginal	0.08	0.164
Caesarean	1.00	
***Presentation***		
Vertex	0.14	0.176
Breech or transverse	1.00	
***Labor pain***		
Present	0.31	< 0.001
Absent	1.00	

*SGA* Small-for-gestational age, *LGA* Large-for-gestational age, *AGA* Appropriate-for-gestational age

Parity and gestational age were not associated with HC changes (Table 2). The researchers further analyzed only newborns who had a HC < P3 at birth or within 24 h of birth. Being symmetrical SGA was the single associated factor of microcephaly at DOL3 (data not shown).

## Discussion

This study compared HC measured at birth or within 24 h of birth to DOL3 as well as assessed mother- and infant-specific factors that affected HC differences between these two time points. The researchers found that the mean HC on DOL3 was significantly greater than HC at birth or within 24 h of birth. In this study’s cohort of 1131 newborns, 32 had microcephaly at birth or within 24 h of birth, but only seven (22.0%) were classified as microcephalic by DOL3. This supports the use of serial HC measurements to accurately classify congenital microcephaly.

Minimal changes in HC can lead to misclassifying infants as microcephalic instead of normocephalic. While a difference of 0.17 cm in HC may not appear like much, the clinical impact associated with the ability to classify whether a newborn is microcephalic is far greater for neonates and their families. The mean increase of HCs between DOL3 and at birth was lower than the mean increase of 0.39 cm reported in a previous study [6], which compared HCs of 499 newborns measured immediately after birth and on DOL3. The researchers observed similar proportions of newborns with vertex presentations between this study and Klarić et al.’s (95.0% vs 97.0%). However, this study had a higher proportion of C-sections (48.8% compared with their 20.4%). Newborns delivered by C-sections were found to have smaller HC, and newborns delivered vaginally larger HC in the seventh day of life compared with their respective HCs at birth [2]. The greater proportion of newborns delivered by C-section in this study’s cohort may explain the relatively small difference in HC observed between these two time points [2]. This study also supports previous findings that SGA was a significant predictor of increased HC at DOL3 [2].

Interestingly, while SGA was an associated factor with increased HC by the time of newborns’ discharge, being SGA also was associated with persistent microcephaly at discharge among those with microcephaly at birth. The researchers postulated that these results were related to underlying causes of SGA in newborns. The etiologies of SGA are multifactorial and affected by maternal factors (most commonly, uteroplacental factors) as well as fetal factors or genetic disorders. Some factors may cause different in-utero fetal environments and postnatal growth catch-up abilities while others influence underlying genetics and cause persistent microcephaly. Compared with appropriate-for-gestational age (AGA) and large-for-gestational age (LGA) newborns, SGA newborns were found to have different body composition (i.e., lower body fat) as well as different anthropometric changes (i.e., less postnatal weight loss and faster catch-up growth rate for both body weight and length). The researchers hypothesized that postnatal HC in SGA may follow the same catch-up pattern. However, newborns’ body weight on DOL3 was not collected. Thus, the researchers were unable to describe and compare these postnatal changes [7, 8]. In addition, the sample size was too small to make any conclusive predictions regarding these different outcomes. This warrants further investigation.

Another factor associated with increased HC at discharge was labor pain. This change resulted from a natural adaptation of skull molding for smooth vaginal delivery. A photographic study demonstrated that newborns of primiparous mothers had significantly higher degrees of molding than those born by multiparous mothers [9]. This study’s findings further support this postnatal unmolding effect, as infants born to primiparous mothers had a greater increased difference in HC compared with multiparous mothers. However, these results were not statistically significant.

The HC at birth was lower than the cut-off for gestational age among the 25 infants classified as microcephalic, but later normocephalic by DOL3 – with a mean increased HC of 0.55 (range: 0.1–1.2) cm. Upon remeasurement, four newborns had HCs in the third to tenth percentiles and 21 had HCs in the tenth to 50^th^ percentiles by DOL3. All were classified as normocephalic at discharge. These findings may be from the unmolding process after birth or measurement errors. The researchers attempted to minimize such errors through standardized training. As noted previously, regardless of the simplicity of HC measurements as well as training and standardization efforts, technical errors may still occur. In a rigorously standardized INTERGROWTH study, HC measurement errors ranged from 0.3–0.4 cm [10]. Another study that evaluated the reliability and validity of parental measurements reported measurement errors of up to 0.6 cm [11]. This again underscores the importance of serial HC measurements and clinical follow-ups after discharge.

This study had some limitations. First, the researchers were unable to classify microcephaly based on prenatal fetal ultrasound reports. The Royal Thai College of Obstetricians and Gynecologists recommends that all pregnant Thai women have at least one fetal ultrasound performed during their second trimester (18–22 weeks of gestation) [12]. Most of the mothers in this study had their second trimester fetal ultrasound. None were classified to have microcephaly. However, microcephaly cases that appeared later during pregnancy may not be detected by prenatal ultrasound, as follow-up ultrasounds are not routinely performed unless abnormalities are detected during the second trimester. Second, while the researchers followed up on eight out of ten newborns with microcephaly at discharge and all 32 newborns with microcephaly at birth, they did not collect long-term outcomes of these infants.

It is general practice to have only a single HC measurement at birth or within 24 h after birth. This study demonstrated that 78.1% of newborns initially classified as microcephalic at birth were later normocephalic by DOL3 and 30% of newborns microcephalic on DOL3 were initially normocephalic at birth or within 24 h after birth. These newborns could have been misclassified as microcephalic or normocephalic had the decision been based solely on HC measurements at birth. This would have hindered subsequent evaluations and therapies.

## Conclusions

Serial HC measurements at birth and DOL3 or discharge are crucial to ensure accurate assessments of changes in anthropometry of newborns and avoid unnecessary investigations, misclassifications, and parental distress. This further highlights the importance of postnatal serial HC measurements in low- and middle-income countries, where prenatal diagnosis of microcephaly is not always feasible during real-life practice.

## Data Availability

The datasets used and/or analyzed during the current study are available from the corresponding author on reasonable request.

## Abbreviations

HC: Head circumference
SGA: Small-for-gestational age
LGA: Large-for-gestational age
LMP: Last menstrual period
ICC: Interclass correlation coefficient
DOL3: 3 days of life
P3: Third percentile
CMV: Cytomegalovirus
CI: Confidence interval
SD: Standard deviation
WHO: World Health Organization
